# Luteolin mitigates oxidative stress and multi-organ impairment in a propionic acid-induced rodent model of autism

**DOI:** 10.3389/fnut.2025.1583119

**Published:** 2025-05-27

**Authors:** Arwa Ishaq Abdulmalik Khayyat, Altaf N. Alabdali, Mona Alonazi, Areej Ali Alzahrani, Eman Al-Shehri, Abir Ben Bacha

**Affiliations:** Department of Biochemistry, College of Science, King Saud University, Riyadh, Saudi Arabia

**Keywords:** autism spectrum disorder, luteolin, oxidative stress, leaky gut, liver, kidney

## Abstract

**Background/objectives:**

Oxidative stress, organ impairments, and gastrointestinal abnormalities are the most common systemic dysfunctions that accompanied the neurodevelopmental condition, Autism Spectrum Disorder (ASD). Emerging evidence suggests that increased propionic acid (PPA) levels contribute to ASD pathophysiology through oxidative stress, neuroinflammation and disruption of the gut-liver-brain axis. Thanks to its strong anti-inflammatory and antioxidant potencies, luteolin, has shown to be promising in alleviating these effects. This study investigated the therapeutic and protective effects of luteolin in a PPA-induced rodent model of ASD by assessing oxidative stress, intestinal permeability, and liver and kidney dysfunction biomarkers.

**Methods:**

Fifty young male albino rats were divided into five groups: control, PPA-treated, luteolin-treated, therapeutic (PPA followed by luteolin), and protective (luteolin followed by PPA). Oxidative stress markers (GSH, lipid peroxides, GST, SOD, and catalase), serum zonulin, liver enzymes (ALT, AST, ALP) and renal function markers (urea nitrogen, creatinine) were investigated. ROC analysis evaluated the diagnostic potential of these biomarkers, while Spearman correlation analysis explored interrelationships among parameters.

**Results:**

PPA administration significantly reduced antioxidant defenses, including GSH, GST, SOD, and catalase, while increasing lipid peroxidation and inducing hepatic and renal dysfunction, as evidenced by elevated ALT, AST, ALP, urea nitrogen, and creatinine levels, along with increased zonulin levels. Luteolin intervention effectively reversed these alterations by restoring antioxidant capacity, lowering zonulin levels, and improving liver and kidney function. ROC analysis demonstrated high diagnostic accuracy (AUC = 1.000) for oxidative stress and organ dysfunction markers in the PPA-treated group, while luteolin treatment significantly enhanced biomarker sensitivity and specificity. Spearman correlation analysis revealed strong negative correlations between antioxidants and oxidative stress markers (*p* < 0.001) and positive correlations between zonulin and liver/kidney dysfunction indicators (*p* < 0.001), further confirming the systemic impact of PPA.

**Conclusion:**

Luteolin effectively alleviated oxidative stress, restored antioxidant defenses, and enhanced liver, kidney, and intestinal barrier functions in a PPA-induced ASD model. These findings underscored its therapeutic potential as a natural intervention for ASD-related systemic dysfunctions. Further clinical studies are needed to evaluate its translational applicability in ASD management.

## 1 Introduction

Repetitive behaviors as well as impairments in communication skills and social interaction are the main characteristics of the neurodevelopmental disorder, autism spectrum disorder (ASD) (1, 2). Globally, ASD impacts about 1 in 54 children, with a higher prevalence among males (3). In addition to its hallmark symptoms, ASD is often accompanied by systemic dysfunctions, including oxidative stress, gastrointestinal (GI) issues and organ-specific abnormalities. Genetic predispositions, environmental effects and gut microbiota (GM) abnormalities are all involved in its pathophysiology (4). ASD is frequently associated with heightened oxidative stress, which contributes to neuroinflammation and behavioral disturbances (5). Altered GM and increased intestinal permeability are typical co-morbidities associated with gut-brain axis dysfunction in ASD (4, 6). Additionally, several researchers are beginning to view autism as a systemic disorder with a potential impact on various organs and physiological system, including the liver (7, 8). A recent study by Ahmed et al. (8) demonstrated liver dysfunction and elevated liver enzymes in autistic children compared to controls. Additionally, it has been discovered that children with autism spectrum disorder have greater levels of bacterial metabolites, which have been linked to both acute renal damage and chronic kidney disease (9, 10).

Propionic acid (PPA) is a short-chain fatty acid naturally found in dairy products such as milk, yogurt and cheese (11, 12). It is mainly produced in the colon when GM ferment undigested food. Once absorbed into the bloodstream, PPA can reach adipose tissue, where it lowers plasma fatty acid levels by inhibiting lipolysis, promoting lipogenesis in fat tissues and suppressing fatty acid synthesis in the liver (13). While PPA is crucial for normal immune and physiological functions, elevated levels can disrupt these processes and lead to adverse effects (14). PPA has the ability to cross barriers in the gut, liver, blood, and brain, allowing it to reach the central nervous system (15). Within the brain, PPA penetrates cell membranes and accumulates inside, causing the cells to become more acidic (16, 17). This acidification disrupts neurotransmitter release, potentially affecting neuronal communication and behavior (18, 19). PPA is also implicated in metabolic disturbances, including inhibition of ureagenesis, disruption of fatty acid metabolism (20), and induction of oxidative stress (21, 22). Its ability to traverse the gut-liver-brain axis facilitates systemic effects, contributing to liver dysfunction, inflammation, and oxidative damage (15).

Medicinal plants are rich in natural compounds that have been widely studied for their pharmacological effects on various conditions, including cancer, inflammation, cardiovascular, and neurodegenerative disorders (23, 24). Flavonoids, key bioactive compounds derived from plants, play essential roles in plant defense and offer a range of health benefits, including anticoagulant, anti-inflammatory, anti-cancer, antimicrobial, and antidepressant effects (25, 26). Their antioxidant properties largely contribute to these benefits. Indeed, the phenolic structure of flavonoids helps neutralizing free radicals and mitigate oxidative stress by decreasing lipid peroxidation and enhancing antioxidant enzymes such as glutathione peroxidase (GPx), catalase and superoxide dismutase (SOD) (27, 28).

Luteolin (3,4,5,7-tetrahydroxyflavone) is a natural flavonoid found in various foods such as carrots, apples, cabbage, and certain medicinal plants. It exhibits a range of pharmacological effects, including anti-cancer, anti-inflammatory, and neuroprotective properties (29). The structure of luteolin is directly linked to its antioxidant capacity and its ability to resist oxidation through metal ion chelation (30–33). Studies have shown that luteolin reduces oxidative stress, regulates inflammatory pathways and restores the integrity of the gut-liver-kidney axis. Specifically, luteolin provides renoprotective benefits, protecting against renal damage caused by ischemia, nephrotoxic drugs, and sepsis (34–36). The co-administration of this bioactive flavonoid as part of preoperative nutrition has been shown to reduce ischemia–reperfusion injury and minimize intestinal apoptosis (37). Luteolin is also an attractive candidate for developing liver-protective drugs. It targets proinflammatory interleukin (IL)-1 and IL-18 pathways, significantly alleviating inflammation and steatosis while restoring normal levels of alanine transaminase (ALT), aspartate transaminase (AST), and maintaining a proper balance between reactive oxygen species and antioxidant enzymes (38). In addition, luteolin has demonstrated therapeutic potential in neurological disorders. A clinical study found that a dietary supplement containing luteolin significantly improved social interaction in children with ASDs (39). More recent research has further confirmed luteolin’s promise as a therapeutic option for reducing ASD symptoms (40). In the present study, the effect of luteolin was investigated in a PPA-induced ASD rat model, focusing on gut leakiness, liver and renal dysfunctions, as well as oxidative stress.

## 2 Materials and methods

### 2.1 Animals and experimental design

Animal experiment was carried out in the Experimental Surgery and Animal Laboratory at the College of Medicine, King Saud University, Riyadh, Saudi Arabia. 50 young male albino rats (80–100 g, 3 weeks old) were housed in cages measuring 40 × 35 × 20 cm^3^ under controlled environmental conditions, with a temperature maintained at 21 ± 1°C and a 12-h light/dark cycle (lights on at 9:00 AM and off at 9:00 PM) and an unrestricted access to water and standard laboratory feed pellets. The experimental procedures were approved by the Ethics Committee for Animal Research at King Saud University, Riyadh (Approval No. KSU-SE-23-26).

The animals were randomly assigned to five groups, each consisting of 10 rats: Group I (Control group): received phosphate-buffered saline (PBS) only. Group II (PPA-intoxicated group): administered PPA at a dose of 250 mg/kg daily for 3 days. Group III (Luteolin-treated group): given luteolin at a dose of 50 mg/kg per day for 27 days. The 50 mg/kg dose of luteolin was selected based on previous studies showing its neuroprotective and anti-inflammatory effects in rats, including models of neurotoxicity (41) and inflammation (42), supporting its use in the PPA-induced ASD model. Group IV (Therapeutic group): received PPA (250 mg/kg daily for 3 days), followed by luteolin (50 mg/kg per day for 27 days). Group V (Protective group): pre-treated with luteolin (50 mg/kg per day for 27 days), followed by PPA administration (250 mg/kg daily for 3 days).

### 2.2 Serum and tissue collection

On day 28, blood samples were collected via direct cardiac puncture into plain tubes without anticoagulant. Serum was separated by centrifugation at 3,000 rpm at 4°C for 10 min.

Meanwhile, the liver and kidney were carefully excised, washed, and sliced into ~1 g fragments. The tissue samples were homogenized in 10 mL of double-distilled water for 3 min using a Teflon homogenizer (Merck KGaA, Darmstadt, Germany). The homogenates were then centrifuged at 4,000 g for 20 min to remove debris. Both tissue homogenates and serum samples were stored at −80°C until further use.

### 2.3 Biochemical analysis

A panel of selected biochemical markers was measured across all study groups. Oxidative stress biomarkers, including glutathione (GSH) (43), lipid peroxides (44), glutathione-S-transferase (GST) (45), SOD (46), and catalase (47), were analyzed in serum, liver, and kidney tissue homogenates.

Serum zonulin (Cat No. MBS2606662) and urea nitrogen (Cat No. MAK006) were quantified using ELISA kits from MyBioSource and Sigma-Aldrich, respectively, following the manufacturers’ instructions. Additionally, alanine aminotransferase (ALT, Cat No. EL07-150), aspartate aminotransferase (AST, Cat No. EL15-150), alkaline phosphatase (ALP, Cat No. EL04L-150), and creatinine (Cat No. EL33K-1000) levels were assessed in serum samples using colorimetric kits from United Diagnostics Industry, Dammam, KSA, according to the manufacturers’ protocols.

All measurements were performed in triplicate, with the mean of three readings calculated.

### 2.4 Statistical analysis

Statistical analysis was conducted using SPSS software (SPSS Inc., Chicago, IL, USA). Data were expressed as mean ± SD. One-way ANOVA and Kruskal-Wallis tests assessed group differences, followed by the Least Significant Difference (LSD) or Mann–Whitney tests for multiple comparisons. Receiver Operating Characteristic (ROC) curve analysis evaluated the diagnostic performance of biochemical parameters, reporting Area Under the Curve (AUC), sensitivity and specificity. Parameters with high AUC values were strong biomarker candidates. Spearman correlation analysis explored relationships between biochemical parameters, reporting correlation coefficients (R) and *p* values.

## 3 Results

**Figure 1:**
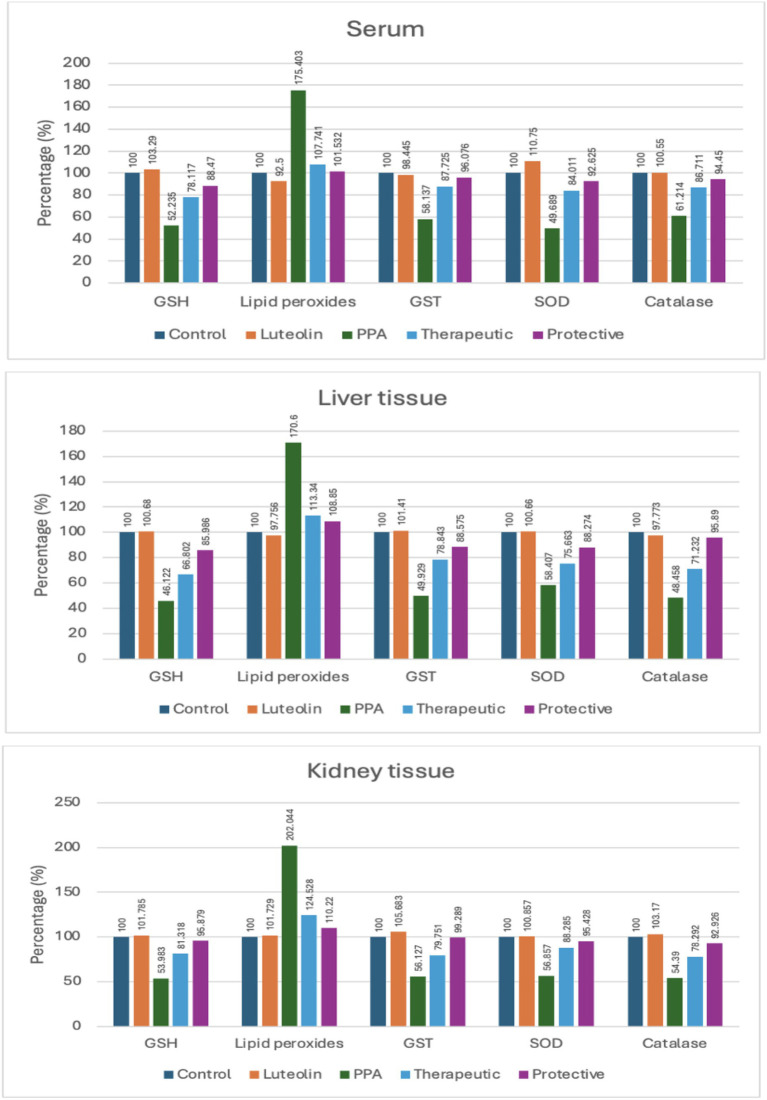
Percent change of oxidative stress biomarkers in serum, liver, and kidney tissues of experimental groups (Control, Luteolin, PPA, Therapeutic and Protective). Data were expressed as percentage relative to control group (set at 100%). GSH, Reduced Glutathione; GST, Glutathione S-Transferase; SOD, Superoxide Dismutase; PPA, Propionic Acid.

Biochemical assessments revealed that PPA exposure significantly disrupted redox homeostasis, marked by a notable reduction in GSH and antioxidant enzyme activities, with a concomitant increase in lipid peroxides across serum, liver, and kidney samples. These findings highlighted oxidative stress as a hallmark of PPA toxicity (Table 1; Figure 1).

**Table 1:** Comparative analysis of oxidative stress biomarkers between different groups.

Parameter	Group	N	Min.	Max.	Mean ± SD	Median	Percent change (Mean)	*p* value
Serum								
GSH (μg/mL)	Control	6	65	77	70.833 ± 4.792^cde^	71	100	<0.001
	Luteolin	6	66	79	73.167 ± 5.115^cde^	74.5	103.29	
	PPA	6	29	45	37 ± 5.44^abde^	37	52.235	
	Therapeutic	6	44	65	55.333 ± 7.941^abc^	56	78.117	
	Protective	6	53	73	62.667 ± 7.34^abc^	63	88.47	
Lipid peroxides (MD μmoles/mL)	Control	6	1.5	2.5	2.067 ± 0.397^c^	2.15	100	<0.001
	Luteolin	6	1.5	2.25	1.912 ± 0.264^c^	1.905	92.5	
	PPA	6	2.9	4.32	3.625 ± 0.551^abde^	3.685	175.403	
	Therapeutic	6	1.73	2.85	2.227 ± 0.433^c^	2.09	107.741	
	Protective	6	1.7	2.45	2.098 ± 0.279^c^	2.12	101.532	
GST (U/mL)	Control	6	47.9	65.8	57.68 ± 6.536^c^	58.49	100	<0.001
	Luteolin	6	45.9	62.8	56.783 ± 5.925^c^	58.1	98.445	
	PPA	6	25.7	40.1	33.533 ± 5.541^abde^	33.55	58.137	
	Therapeutic	6	43.8	61.2	50.6 ± 6.152^c^	50.15	87.725	
	Protective	6	48.1	61.5	55.417 ± 5.562^c^	55.9	96.076	
SOD (U/mL)	Control	6	19.2	28.9	24.183 ± 3.755^cd^	23.95	100	<0.001
	Luteolin	6	22.6	31.1	26.783 ± 3.06^cde^	26.9	110.75	
	PPA	6	9.6	14.5	12.017 ± 1.860^abde^	12.15	49.689	
	Therapeutic	6	16.2	25.5	20.317 ± 3.315^abc^	19.9	84.011	
	Protective	6	18.9	26.2	22.4 ± 2.995^bc^	22.3	92.625	
Catalase (U/dL)	Control	6	4.78	6.37	5.707 ± 0.556^cd^	5.75	100	<0.001
	Luteolin	6	5.07	6.54	5.738 ± 0.590^cd^	5.63	100.55	
	PPA	6	2.72	4.2	3.493 ± 0.624^abde^	3.48	61.214	
	Therapeutic	6	4.05	5.57	4.948 ± 0.569^abc^	5.08	86.711	
	Protective	6	4.23	6.31	5.39 ± 0.771^c^	5.51	94.45	
Liver tissue								
GSH (μg/mL)	Control	6	108	132	122.5 ± 8.596^cde^	124	100	< 0.001
	Luteolin	6	115	130	123.33 ± 5.501^cde^	123.5	100.68	
	PPA	6	49	63	56.5 ± 5.431^abde^	57	46.122	
	Therapeutic	6	73	89	81.833 ± 6.112^abce^	81.5	66.802	
	Protective	6	93	125	105.33 ± 12.484^abcd^	102	85.986	
Lipid peroxides (MD μmoles/mL)	Control	6	1.23	1.59	1.411 ± 0.130^cd^	1.4	100	< 0.001
	Luteolin	6	1.17	1.59	1.38 ± 0.163^cde^	1.395	97.756	
	PPA	6	2.23	2.55	2.408 ± 0.12^abde^	2.415	170.6	
	Therapeutic	6	1.43	1.72	1.6 ± 0.104^abc^	1.615	113.34	
	Protective	6	1.36	1.68	1.537 ± 0.121^bc^	1.56	108.85	
GST (U/mL)	Control	6	107	135	118.167 ± 10.18^cde^	116.5	100	< 0.001
	Luteolin	6	108	134	119.833 ± 9.621^cde^	119	101.41	
	PPA	6	46	75	59 ± 10.695^abde^	56.5	49.929	
	Therapeutic	6	83	104	93.167 ± 7.731^abc^	94	78.843	
	Protective	6	91	119	104.67 ± 10.289^abc^	104.5	88.575	
SOD (U/mL)	Control	6	66	85	75.333 ± 7.527^cde^	75	100	< 0.001
	Luteolin	6	65	86	75.833 ± 8.612^cde^	75.5	100.66	
	PPA	6	33	54	44 ± 8.05^abde^	44.5	58.407	
	Therapeutic	6	47	63	57 ± 6.033^abce^	58.5	75.663	
	Protective	6	59	75	66.5 ± 6.411^abcd^	66.5	88.274	
Catalase (U/dL)	Control	6	88	112	97.333 ± 9.331^cd^	94.5	100	< 0.001
	Luteolin	6	85	106	95.167 ± 8.329^cd^	95.5	97.773	
	PPA	6	39	57	47.167 ± 6.432^abde^	47	48.458	
	Therapeutic	6	59	81	69.333 ± 7.554^abce^	68.5	71.232	
	Protective	6	78	108	93.333 ± 11.093^cd^	95.5	95.89	
Kidney tissue								
GSH (μg/mL)	Control	6	107	131	121.33 ± 8.959^cd^	122.5	100	< 0.001
	Luteolin	6	102	139	123.5 ± 12.787^cd^	125	101.785	
	PPA	6	53	77	65.5 ± 8.849^abde^	65.5	53.983	
	Therapeutic	6	86	112	98.667 ± 9.266^abce^	98	81.318	
	Protective	6	99	132	116.33 ± 12.127^cd^	117	95.879	
Lipid peroxides (MD μmoles/mL)	Control	6	0.82	1.35	1.06 ± 0.176^cd^	1.055	100	< 0.001
	Luteolin	6	0.93	1.25	1.078 ± 0.122^cd^	1.085	101.729	
	PPA	6	1.9	2.55	2.142 ± 0.244^abde^	2.075	202.044	
	Therapeutic	6	1.19	1.45	1.32 ± 0.092^abc^	1.31	124.528	
	Protective	6	1.01	1.31	1.168 ± 0.112^c^	1.17	110.22	
GST (U/mL)	Control	6	83	108	93.833 ± 9.786^cd^	92.5	100	< 0.001
	Luteolin	6	89	112	99.167 ± 10.206^cd^	95	105.683	
	PPA	6	45	59	52.667 ± 5.316^abde^	53.5	56.127	
	Therapeutic	6	65	83	74.833 ± 6.853^abce^	76	79.751	
	Protective	6	83	102	93.167 ± 7.250^cd^	94	99.289	
SOD (U/mL)	Control	6	49	66	58.333 ± 5.887^c^	59	100	< 0.001
	Luteolin	6	51	68	58.833 ± 6.493^cd^	58.5	100.857	
	PPA	6	26	40	33.167 ± 5.231^abde^	34.5	56.857	
	Therapeutic	6	44	61	51.5 ± 6.025^bc^	51	88.285	
	Protective	6	47	63	55.667 ± 5.922^c^	56.5	95.428	
Catalase (U/dL)	Control	6	62	77	68.333 ± 5.278^cd^	67.5	100	< 0.001
	Luteolin	6	64	77	70.5 ± 4.764^cd^	69.5	103.17	
	PPA	6	31	44	37.167 ± 4.792^abde^	36.5	54.39	
	Therapeutic	6	47	67	53.5 ± 7.204^abce^	51.5	78.292	
	Protective	6	55	75	63.5 ± 7.61^cd^	61.5	92.926	

Table 1 describes One-way ANOVA Test between different groups with Multiple Comparisons (LSD test) within the entire groups for each parameter.

^a^ Describes significant difference between the group and the (Control group) at significant level (0.05).

^b^ Describes significant difference between the group and the (Luteolin-Treated group) at significant level (0.05).

^c^ Describes significant difference between the group and the (PPA-Treated group) at significant level (0.05).

^d^ Describes significant difference between the group and the (Therapeutic group) at significant level (0.05).

^e^ Describes significant difference between the group and the (Protective group) at significant level (0.05).

Luteolin treatment whether administered as a preventive or therapeutic intervention effectively restored antioxidant balance. GSH levels, suppressed by PPA, were markedly elevated in luteolin-treated groups, with the protective protocol demonstrating slightly superior outcomes. Similarly, lipid peroxidation, significantly elevated by PPA, was remarkably reduced following luteolin administration, nearing control levels, particularly in the protective group (Table 1; Figure 1).

The activities of key enzymatic antioxidants, including GST, SOD and catalase, were also significantly impaired by PPA. Interestingly, luteolin co-administration restored these activities in both liver and kidney tissues, with the protective regimen showing greater efficacy in normalizing catalase activity (Table 1; Figure 1).

**Figure 2:**
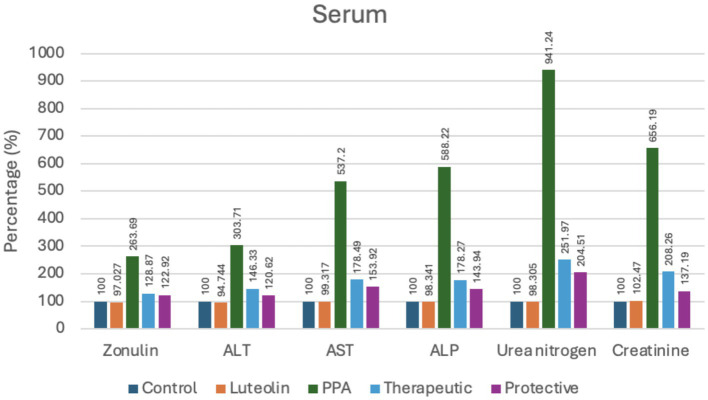
Percent change of serum markers indicating intestinal permeability, liver and kidney function in experimental groups (Control, Luteolin, PPA, Therapeutic and Protective). Data were expressed as percentage relative to control group (set at 100%). ALT, Alanine Aminotransferase; AST, Aspartate Aminotransferase; ALP, Alkaline Phosphatase; PPA, Propionic Acid.

In addition to oxidative stress markers, zonulin levels a biomarker of intestinal permeability—were markedly increased by PPA but significantly reduced in both luteolin-treated groups. These results underscored luteolin’s ability to maintain intestinal barrier integrity (Table 2; Figure 2).

**Table 2:** Comparative analysis of serum zonulin, liver enzymes and kidney function biomarkers between different groups.

Parameter	Group	N	Min.	Max.	Mean ± SD	Median	Percent change (Mean)	*p* value
Zonulin (ng/mL)	Control	6	0.68	0.91	0.785 ± 0.087^cde^	0.78	100	<0.001
	Luteolin	6	0.68	0.84	0.761 ± 0.063^cde^	0.75	97.027	
	PPA	6	1.85	2.32	2.07 ± 0.18^abde^	2.05	263.69	
	Therapeutic	6	0.87	1.15	1.011 ± 0.107^abc^	1.02	128.87	
	Protective	6	0.83	1.14	0.965 ± 0.114^abc^	0.94	122.92	
ALT (U/L)	Control	6	39	55	46.616 ± 5.995^cde^	46.5	100	<0.001
	Luteolin	6	38	51	44.167 ± 4.875^cde^	44	94.744	
	PPA	6	127.5	154	141.58 ± 9.178^abde^	142	303.71	
	Therapeutic	6	60.5	77.5	68.217 ± 6.284^abce^	68	146.33	
	Protective	6	48.7	63.1	56.233 ± 5.522^abcd^	56.75	120.62	
AST (U/L)	Control	6	43	55	48.833 ± 4.665^cde^	48.5	100	<0.001
	Luteolin	6	44	54	48.5 ± 4.135^cde^	47.5	99.317	
	PPA	6	237	302	262.33 ± 22.205^abde^	259	537.2	
	Therapeutic	6	69	101	87.167 ± 12.656^abc^	90.5	178.49	
	Protective	6	66	85	75.167 ± 7.223^abc^	75	153.92	
ALP (U/L)	Control	6	90	113	100.5 ± 8.264^cde^	100	100	<0.001
	Luteolin	6	91	105	98.833 ± 4.875^cde^	99.5	98.341	
	PPA	6	527	645	591.167 ± 48.11^abde^	593.5	588.22	
	Therapeutic	6	129	264	179.167 ± 49.507^abc^	167.5	178.27	
	Protective	6	113	179	144.667 ± 25.781^abc^	146	143.94	
Urea nitrogen (mg/dL)	Control	6	24	35	29.5 ± 4.037^cde^	29	100	<0.001
	Luteolin	6	23	34	29.0 ± 4.0^cde^	29.5	98.305	
	PPA	6	225	337	277.67 ± 40.623^abde^	267.5	941.24	
	Therapeutic	6	52	93	74.333 ± 14.583^abc^	75	251.97	
	Protective	6	51	72	60.333 ± 8.756^abc^	59.5	204.51	
Creatinine (mg/dL)	Control	6	8	12	10.083 ± 1.428^cd^	10.25	100	<0.001
	Luteolin	6	9	12	10.333 ± 1.080^cd^	10.25	102.47	
	PPA	6	55	77	66.167 ± 8.305^abde^	66.5	656.19	
	Therapeutic	6	17	26	21.0 ± 3.464^abce^	20.5	208.26	
	Protective	6	10	18	13.833 ± 3.060^cd^	14	137.19	
						

Table 2 describes One-way ANOVA Test between different groups with Multiple Comparisons (LSD test) within the entire groups for each parameter.

^a^ Describes significant difference between the group and the (Control group) at significant level (0.05).

^b^ Describes significant difference between the group and the (Luteolin-Treated group) at significant level (0.05).

^c^ Describes significant difference between the group and the (PPA-Treated group) at significant level (0.05).

^d^ Describes significant difference between the group and the (Therapeutic group) at significant level (0.05).

^e^ Describes significant difference between the group and the (Protective group) at significant level (0.05).

Liver function biomarkers (ALT, AST and ALP) and renal function markers (urea nitrogen and creatinine) were also severely elevated following PPA exposure, indicating multi-organ impairment. Notably, luteolin administration substantially ameliorated these alterations, with the protective approach yielding values closer to baseline compared to the therapeutic group (Table 2; Figure 2).

**Figure 3:**
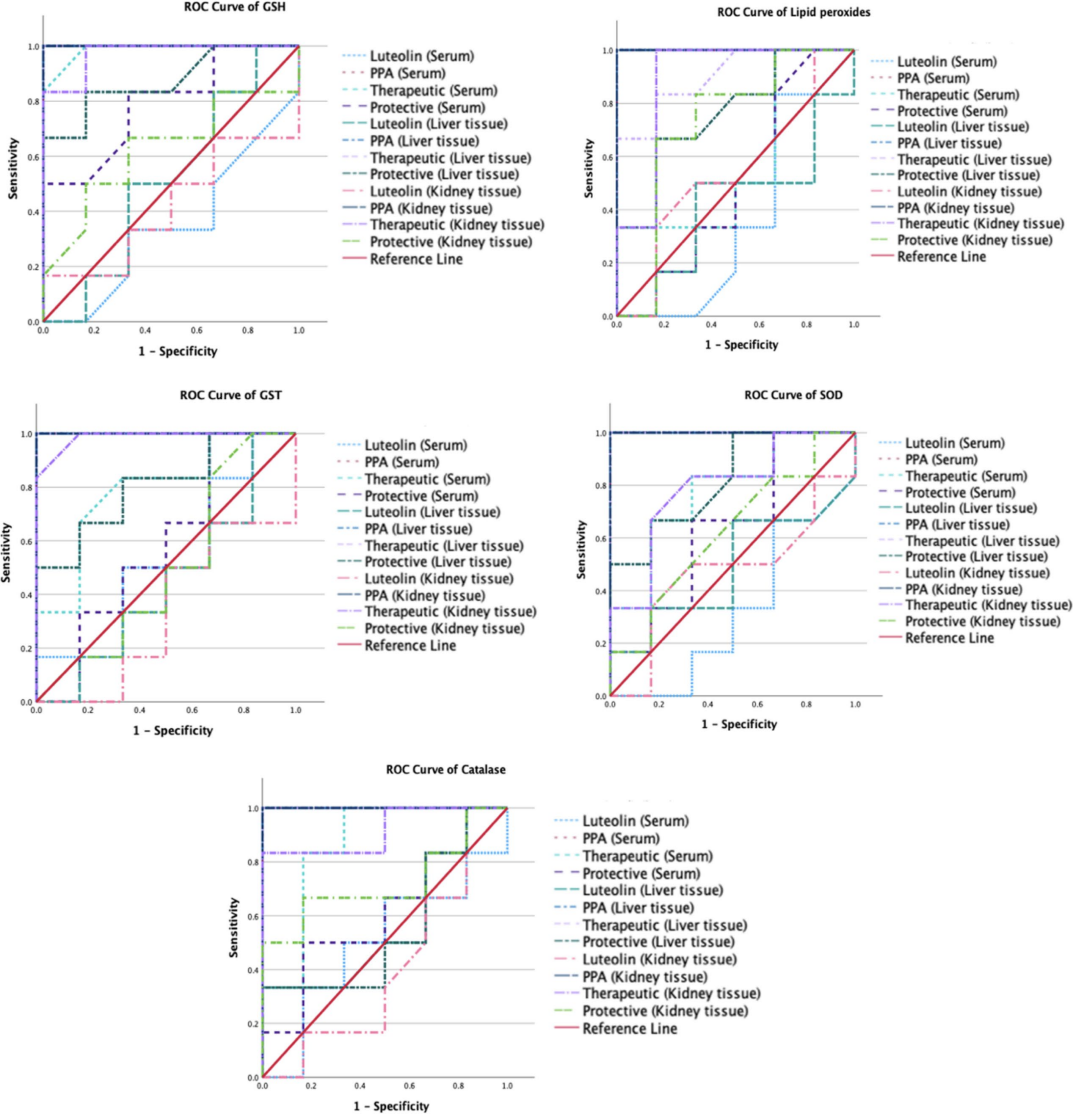
ROC curves of the measured oxidative stress biomarkers in different groups.

**Figure 4:**
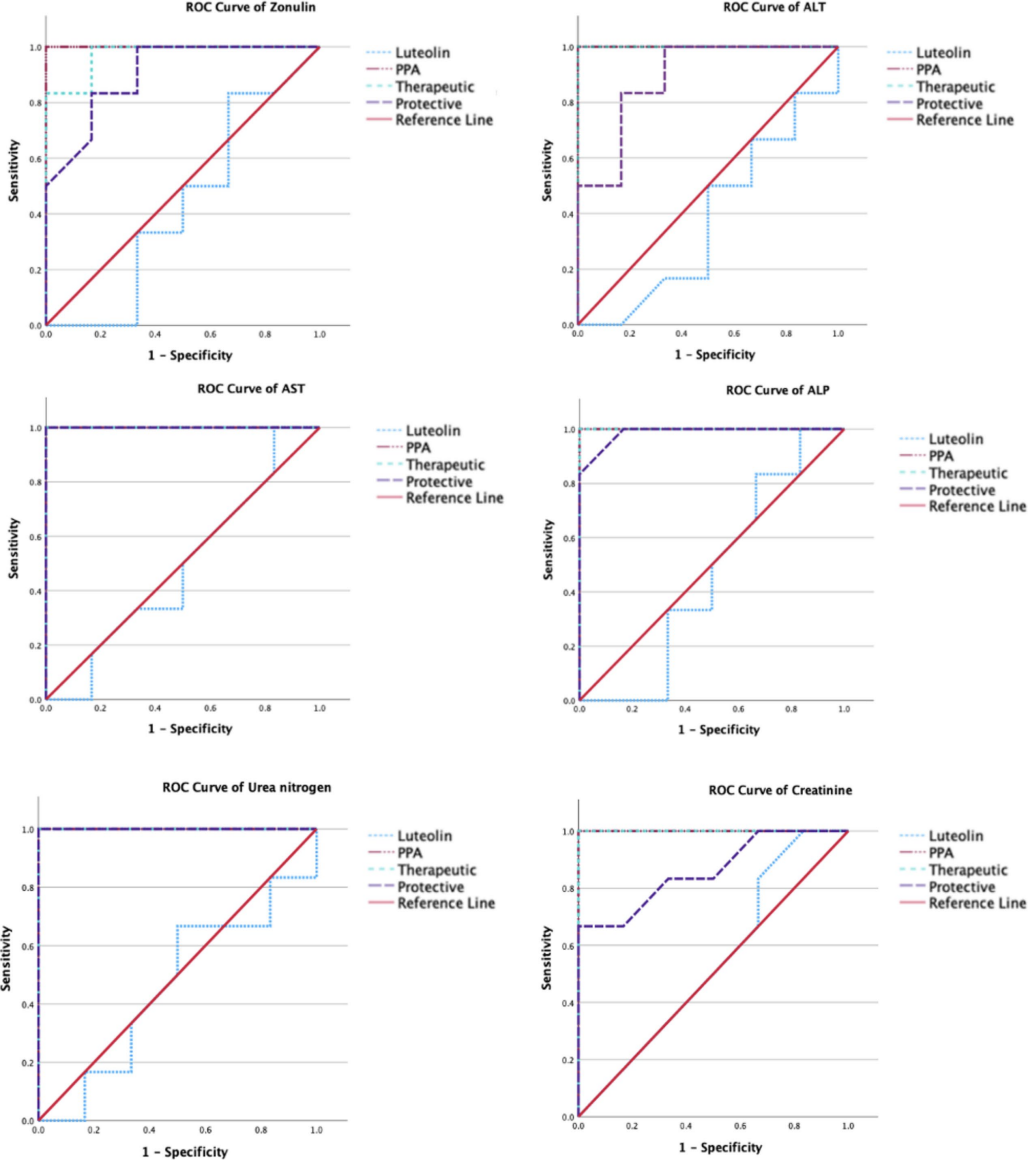
ROC curves of several biomarkers of intestine (Zonulin), liver (ALT, AST & ALP), and renal (urea and creatinine) functioning in the different groups.

The ROC analysis was conducted to evaluate the diagnostic performance of multiple biochemical markers across all experimental groups (Tables 3, 4; Figures 3,4). In the PPA-treated group, all biomarkers exhibited outstanding diagnostic accuracy, with AUC values of 1.0, confirming the significant biochemical and physiological disturbances induced by PPA. In contrast, the luteolin-only group showed low to moderate diagnostic performance across most parameters, with AUC values generally below 0.7, indicating limited predictive power when administered without PPA exposure.

**Table 3:** ROC analysis of the oxidative stress biomarkers in the different groups.

Parameter	Group	AUC	Cut-off value	Sensitivity %	Specificity %	*p* value	95% CI
Serum							
GSH	Luteolin	0.347	64	0.00%	100.00%	0.351	0.026–0.668
	PPA	1	55	100.00%	100.00%	0	1.000–1.000
	Therapeutic	0.986	63.5	83.30%	100.00%	0	0.932–1.040
	Protective	0.792	62.5	50.00%	100.00%	0.03	0.528–1.055
Lipid peroxides	Luteolin	0.361	1.74	83.30%	33.30%	0.424	0.021–0.702
	PPA	1	2.7	100.00%	100.00%	0	1.000–1.000
	Therapeutic	0.583	2.575	33.30%	100.00%	0.64	0.234–0.933
	Protective	0.5	1.79	83.30%	33.30%	1	0.152–0.848
GST	Luteolin	0.528	46.9	16.70%	100.00%	0.875	0.182–0.874
	PPA	1	44	100.00%	100.00%	0	1.000–1.000
	Therapeutic	0.792	51.75	66.70%	83.30%	0.033	0.524–1.060
	Protective	0.583	62.1	100.00%	33.30%	0.633	0.241–0.926
SOD	Luteolin	0.319	18.2	0.00%	100.00%	0.26	0.005–0.634
	PPA	1	16.85	100.00%	100.00%	0	1.000–1.000
	Therapeutic	0.778	20.7	66.70%	83.30%	0.0461	0.505–1.051
	Protective	0.639	23.15	66.70%	66.70%	0.407	0.311–0.967
Catalase	Luteolin	0.5	5.355	33.30%	83.30%	1	0.153–0.847
	PPA	1	4.49	100.00%	100.00%	0	1.000–1.000
	Therapeutic	0.861	5.425	83.30%	83.30%	0.002	0.634–1.088
	Protective	0.611	5.36	50.00%	83.30%	0.513	0.278–0.944
Liver tissue							
GSH	Luteolin	0.514	122.5	50.00%	66.70%	0.937	0.168–0.860
	PPA	1	85.5	100.00%	100.00%	0	1.000–1.000
	Therapeutic	1	98.5	100.00%	100.00%	0	1.000–1.000
	Protective	0.875	106.5	66.70%	100.00%	0	0.666–1.084
Lipid peroxides	Luteolin	0.417	1.465	50.00%	66.70%	0.633	0.074–0.759
	PPA	1	1.91	100.00%	100.00%	0	1.000–1.000
	Therapeutic	0.903	1.595	66.70%	100.00%	0	0.728–1.078
	Protective	0.764	1.53	66.70%	83.30%	0.063	0.486–1.042
GST	Luteolin	0.444	134.5	100.00%	16.70%	0.75	0.103–0.786
	PPA	1	91	100.00%	100.00%	0	1.000–1.000
	Therapeutic	1	105.5	100.00%	100.00%	0	1.000–1.000
	Protective	0.806	104	50.00%	100.00%	0.019	0.550–1.061
SOD	Luteolin	0.486	65.5	16.70%	100.00%	0.938	0.137–0.835
	PPA	1	60	100.00%	100.00%	0	1.000–1.000
	Therapeutic	1	64.5	100.00%	100.00%	0	1.000–1.000
	Protective	0.819	65.5	50.00%	100.00%	0.01	0.576–1.063
Catalase	Luteolin	0.569	87.5	33.30%	100.00%	0.693	0.224–0.914
	PPA	1	72.5	100.00%	100.00%	0	1.000–1.000
	Therapeutic	1	84.5	100.00%	100.00%	0	1.000–1.000
	Protective	0.556	85.5	33.30%	100.00%	0.755	0.206–0.905
Kidney tissue							
GSH (μg/mL)	Luteolin	0.417	104.5	16.70%	100.00%	0.633	0.074–0.759
	PPA	1	92	100.00%	100.00%	0	1.000–1.000
	Therapeutic	0.972	106	83.30%	100.00%	0	0.889–1.056
	Protective	0.625	115.5	50.00%	83.30%	0.468	0.288–0.962
Lipid peroxides	Luteolin	0.542	1.14	33.30%	83.30%	0.812	0.197–0.886
	PPA	1	1.625	100.00%	100.00%	0	1.000–1.000
	Therapeutic	0.889	1.155	100.00%	83.30%	0	0.673–1.105
	Protective	0.722	1.125	66.70%	83.30%	0.173	0.403–1.042
GST	Luteolin	0.333	82	0.00%	100.00%	0.308	0.013–0.654
	PPA	1	71	100.00%	100.00%	0	1.000–1.000
	Therapeutic	0.986	81.5	83.30%	100.00%	0	0.932–1.040
	Protective	0.5	100.5	83.30%	33.30%	1	0.152–0.848
SOD	Luteolin	0.472	54.5	33.30%	83.30%	0.877	0.122–0.823
	PPA	1	44.5	100.00%	100.00%	0	1.000–1.000
	Therapeutic	0.792	53.5	66.70%	83.30%	0.033	0.524–1.060
	Protective	0.625	48	16.70%	100.00%	0.454	0.298–0.952
Catalase	Luteolin	0.389	61	0.00%	100.00%	0.515	0.055–0.723
	PPA	1	53	100.00%	100.00%	0	1.000–1.000
	Therapeutic	0.917	58.5	83.30%	100.00%	0	0.742–1.091
	Protective	0.722	61	50.00%	100.00%	0.162	0.411–1.034

**Table 4:** ROC analysis of several biomarkers of intestine (Zonulin), liver (ALT, AST & ALP), and renal (urea and creatinine) functioning in the different groups.

Parameter	Group	AUC	Cut-off value	Sensitivity %	Specificity %	*p* value	95% CI
Zonulin	Luteolin	0.431	0.715	83.30%	33.30%	0.693	0.086–0.776
	PPA	1	1.38	100.00%	100.00%	0	1.000–1.000
	Therapeutic	0.972	0.92	83.30%	100.00%	0	0.889–1.056
	Protective	0.903	0.87	83.30%	83.30%	0	0.728–1.078
ALT	Luteolin	0.375	56	0.00%	100.00%	0.458	0.045–0.705
	PPA	1	91.25	100.00%	100.00%	0	1.000–1.000
	Therapeutic	1	57.75	100.00%	100.00%	0	1.000–1.000
	Protective	0.889	51.35	83.30%	83.30%	0	0.697–1.081
AST	Luteolin	0.486	43.5	100.00%	16.70%	0.937	0.143–0.829
	PPA	1	146	100.00%	100.00%	0	1.000–1.000
	Therapeutic	1	62	100.00%	100.00%	0	1.000–1.000
	Protective	1	60.5	100.00%	100.00%	0	1.000–1.000
ALP	Luteolin	0.458	95	83.30%	33.30%	0.816	0.108–0.809
	PPA	1	320	100.00%	100.00%	0	1.000–1.000
	Therapeutic	1	121	100.00%	100.00%	0	1.000–1.000
	Protective	0.986	117	83.30%	100.00%	0	0.932–1.040
Urea nitrogen	Luteolin	0.458	28.5	66.70%	50.00%	0.812	0.115–0.801
	PPA	1	130	100.00%	100.00%	0	1.000–1.000
	Therapeutic	1	43.5	100.00%	100.00%	0	1.000–1.000
	Protective	1	43	100.00%	100.00%	0	1.000–1.000
Creatinine	Luteolin	0.542	9.25	83.30%	33.30%	0.811	0.200–0.884
	PPA	1	33.5	100.00%	100.00%	0	1.000–1.000
	Therapeutic	1	14.5	100.00%	100.00%	0	1.000–1.000
	Protective	0.861	12.5	66.70%	100.00%	0.001	0.644–1.079

Luteolin co-administration in the therapeutic and protective groups substantially enhanced the diagnostic accuracy of numerous markers. In the therapeutic group, several serum and tissue biomarkers such as GSH, ALT, AST, ALP and lipid peroxides exhibited excellent performance (AUC > 0.9), reflecting luteolin’s robust capacity to mitigate PPA-induced biochemical alterations. Similarly, the protective group demonstrated marked improvement in AUC values for key oxidative stress and organ function biomarkers, though to a slightly lesser extent than the therapeutic group. These findings collectively emphasize the potential of luteolin in both preventing and reversing PPA-induced oxidative and functional impairments.

Spearman’s correlation analysis revealed significant interrelationships among biochemical parameters, highlighting the interconnected roles of oxidative stress, intestinal permeability, and liver and kidney function (Table 5). Antioxidants such as GSH, GST, SOD and catalase exhibited strong positive correlations. In contrast, lipid peroxides showed negative correlations with antioxidants. Zonulin displayed a strong negative correlation with antioxidants but was positively correlated with lipid peroxides, ALT and AST. Liver function markers (ALT and AST) were highly correlated with each other and with zonulin, while showing strong negative correlations with antioxidants such as GSH. Similarly, kidney function markers (urea nitrogen and creatinine) were strongly correlated and also positively associated with zonulin. However, they exhibited negative correlations with antioxidants, including GSH.

**Table 5:** Correlations between the measured parameters using Spearman’s Correlation.

Parameters	R (Correlation coefficient)	*p* value
GSH ~ Lipid peroxides	−0.564^**^	0.001
GSH ~ GST	0.605^**^	<0.001
Lipid peroxides ~ GST	0.493^**^	0.006
GSH ~ SOD	0.658^**^	<0.001
Lipid peroxides ~ SOD	−0.542^**^	0.002
GST ~ SOD	0.507^**^	0.004
GSH ~ Catalase	0.616^**^	<0.001
Lipid peroxides ~ Catalase	−0.394^*^	0.031
GST ~ Catalase	0.756^**^	<0.001
SOD ~ Catalase	0.702^**^	<0.001
GSH ~ Zonulin	−0.698^**^	<0.001
Lipid peroxides ~ Zonulin	0.622^**^	<0.001
GST ~ Zonulin	−0.589^**^	<0.001
SOD ~ Zonulin	−0.714^**^	<0.001
Catalase ~ Zonulin	−0.579^**^	<0.001
GSH ~ ALT	−0.756^**^	<0.001
Lipid peroxides ~ ALT	0.572^**^	<0.001
GST ~ ALT	−0.694^**^	<0.001
SOD ~ ALT	−0.807^**^	<0.001
Catalase ~ ALT	−0.703^**^	<0.001
Zonulin ~ ALT	0.873^**^	<0.001
GSH ~ AST	−0.781^**^	<0.001
Lipid peroxides ~ AST	0.614^**^	<0.001
GST ~ AST	−0.526^**^	0.003
SOD ~ AST	−0.779^**^	<0.001
Catalase ~ AST	−0.613^**^	<0.001
Zonulin ~ AST	0.875^**^	<0.001
ALT ~ AST	0.893^**^	<0.001
GSH ~ ALP	−0.763^**^	<0.001
Lipid peroxides ~ ALP	0.648^**^	<0.001
GST ~ ALP	−0.664^**^	<0.001
SOD ~ ALP	−0.675^**^	<0.001
Catalase ~ ALP	−0.695^**^	<0.001
Zonulin ~ ALP	0.830^**^	<0.001
ALT ~ ALP	0.867^**^	<0.001
AST ~ ALP	0.907^**^	<0.001
GSH ~ Urea nitrogen	−0.761^**^	<0.001
Lipid peroxides ~ Urea nitrogen	0.563^**^	<0.001
GST ~ Urea nitrogen	−0.670^**^	<0.001
SOD ~ Urea nitrogen	−0.807^**^	<0.001
Catalase ~ Urea nitrogen	−0.758^**^	<0.001
Zonulin ~ Urea nitrogen	0.883^**^	<0.001
ALT ~ Urea nitrogen	0.907^**^	<0.001
AST ~ Urea nitrogen	0.883^**^	<0.001
ALP ~ Urea nitrogen	0.857^**^	<0.001
GSH ~ Creatinine	−0.811^**^	<0.001
Lipid peroxides ~ Creatinine	0.586^**^	<0.001
GST ~ Creatinine	−0.735^**^	<0.001
SOD ~ Creatinine	−0.694^**^	<0.001
Catalase ~ Creatinine	−0.741^**^	<0.001
Zonulin ~ Creatinine	0.814^**^	<0.001
ALT ~ Creatinine	0.879^**^	<0.001
AST ~ Creatinine	0.834^**^	<0.001
ALP ~ Creatinine	0.858^**^	<0.001
Urea nitrogen ~ Creatinine	0.869^**^	<0.001

* Correlation is significant at the 0.05 level.

** Correlation is significant at the 0.01 level.

## 4 Discussion

This study demonstrated the promising therapeutic and preventive potential of luteolin in mitigating PPA-induced oxidative stress, intestinal barrier dysfunction, and hepatic and renal impairments in a rodent model of ASD. Through biochemical assessments and robust statistical analyses including Spearman correlations and ROC curve evaluations, luteolin showed a strong capacity to restore antioxidant defense mechanisms, reinforce intestinal barrier integrity, and normalize liver and kidney function biomarkers.

Emerging research also underscores the pivotal role of the gut–liver and gut–kidney axes in systemic health. The gut–liver axis facilitates metabolite and immune communication via the portal vein, and its disruption via dysbiosis and increased intestinal permeability can promote hepatic inflammation and oxidative stress (48). Similarly, the gut–kidney axis links microbial metabolites to renal function, with permeability alterations leading to uremic toxin translocation and kidney injury (49). In the current model, PPA-induced permeability may have facilitated harmful microbial product transfer to the liver and kidneys, exacerbating damage. The protective effects of luteolin may thus be partially attributed to its capacity to modulate gut microbiota and enhance intestinal barrier integrity, thereby attenuating pathological processes along these axes (50).

PPA administration lead to increased intestinal permeability, reflected in elevated serum zonulin levels, and widespread oxidative stress, marked by depleted antioxidant enzymes (GSH, GST, SOD, catalase) and increased lipid peroxidation. These results are consistent with previous studies by Kara et al. (51) and Esnafoglu et al. (52), who reported higher zonulin levels in individuals with ASD, indicating a disrupted intestinal barrier. This “leaky gut” state facilitates the entry of microbial products into circulation, exacerbating systemic inflammation and oxidative damage a key mechanism of PPA toxicity (19). Our findings confirmed significantly reduced antioxidant levels and elevated oxidative markers. These results aligned with previous studies reporting oxidative stress in serum (53), liver tissues (15), brain tissues (54, 55), and systemic oxidative stress studies (21, 22).

These oxidative disruptions were strongly correlated with hepatic and renal dysfunction, as reflected in elevated ALT, AST, urea nitrogen, and creatinine, reinforcing previous evidence of PPA-induced multi-organ toxicity (14, 15, 54). However, luteolin intervention, particularly in the protective group, markedly reversed these effects, confirming its potent antioxidant and anti-inflammatory actions. Luteolin treatment significantly improved hepatic oxidative balance likely due to free radical scavenging and upregulation of antioxidant enzymes (27). This finding could be supported by Yao et al. (56) who underlined the hepatoprotective properties of luteolin, especially through the upregulation of antioxidant defenses, including SOD and GSH and the inhibition of oxidative stress markers such as malondialdehyde. This could explain the pronounced recovery observed in liver tissues, where luteolin’s activation of the Nrf2/ARE pathway plays a crucial role (57).

Similarly, kidney tissues responded favorably to luteolin which was consistent with previous studies such as that by Birman et al. (58), where luteolin restored nitric oxide synthase activity and reduced iNOS levels, contributing to renal antioxidative effects. In the current study, both the therapeutic and protective groups restored antioxidant defenses, with the protective group showing greater recovery, supporting luteolin’s role as a potent antioxidant (29). Luteolin’s phenolic structure enhances its free radical scavenging capacity through hydrogen atom donation, reducing oxidative stress markers such as lipid peroxides, as previously reported by Procházková et al. (27) and Wu et al. (28). Both luteolin-treated groups demonstrated a reduction in these markers, restoring near-normal levels, further confirming its hepatoprotective and renoprotective effects (34, 56, 59). Additionally, luteolin’s ability to modulate inflammatory pathways, including IL-1 and IL-18, likely contributes to its protective effects (39).

Elevated zonulin levels in ASD have been associated with greater symptom severity, pointing to a link between intestinal permeability and metabolic disruption (51, 52, 60). In this study, luteolin significantly reduced serum zonulin in both therapeutic and protective groups, suggesting its role in preserving intestinal barrier integrity (50, 61). Interestingly, a significant inverse correlation was found between antioxidant levels (such as GSH and SOD) and serum zonulin, suggesting that oxidative stress contributes directly to intestinal barrier dysfunction. This finding was in line with a recent study by Yuan et al. (62) that demonstrated luteolin’s ability to restore barrier integrity in a Caco-2 cell model by reducing ROS, increasing antioxidant enzyme activity (SOD and GSH), and upregulating tight junction proteins like ZO-1, occludin, and claudin-1. The study also showed that luteolin’s protective effects involve activation of Nrf2 signaling and suppression of ERK and NF-κB/MLCK pathways. These results highlighted the importance of antioxidant defense in modulating intestinal permeability, and supported the use of luteolin as a dietary supplement to preserve gut integrity under oxidative stress conditions.

Correlation analysis further illuminated the underlying pathophysiology, revealing strong negative correlations between oxidative stress markers and antioxidants, and positive correlations with liver and kidney damage parameters (e.g., ALT, AST, creatinine). These findings underscored oxidative stress as a central driver of PPA-induced multi-organ damage and highlighted the importance of antioxidant defense in mitigating systemic toxicity. The antioxidant and protective role of luteolin was well supported by previous research. For instance, Owumi et al. (63) demonstrated that luteolin co-treatment alleviated doxorubicin-induced hepatorenal toxicity in rats by reducing oxidative stress, inflammation, and apoptosis, while improving antioxidant status, tissue integrity, and survival. This reinforces luteolin’s potential as a chemoprotective agent in oxidative stress-related pathologies.

Furthermore, the ROC analysis could provide valuable insights into the diagnostic efficacy of various biomarkers for detecting PPA-induced oxidative stress and organ dysfunction, as well as the therapeutic and protective potential of luteolin. In the PPA group, nearly all biomarkers had AUC = 1.00, indicating severe systemic damage with high diagnostic accuracy (15, 64, 65). Upon luteolin treatment, the therapeutic group exhibited notable improvements. Serum GSH levels, for instance, achieved an AUC of 0.986, with 83.3% sensitivity and 100% specificity, highlighting a significant recovery in antioxidant capacity. Consistent with Ben Bacha et al. (55), luteolin demonstrated neuroprotective and neurotherapeutic properties, effectively counteracting oxidative stress and cognitive impairment. Similarly, liver enzymes (ALT, AST, ALP) and renal function biomarkers (urea nitrogen, creatinine) maintained an AUC of 1.000, indicating complete restoration of liver and kidney functions. These findings aligned with previous research on luteolin’s antioxidative and organ-protective properties. For instance, Yao et al. (56) explored its pharmacological effects and pharmacokinetics in mitigating liver injury, while Diniz et al. (29) highlighted its renoprotective effects against kidney damage.

In the protective group, while some biomarkers, such as AST and urea nitrogen, retained an AUC of 1.000, others, like serum GSH, exhibited reduced sensitivity (50.0%) despite (100%) specificity, resulting in a lower AUC of 0.792. This suggested that preventive luteolin administration provided partial protection against PPA-induced oxidative stress and organ dysfunction. These findings could be supported by previous researches exploring luteolin’s protective effects in oxidative stress models. For instance, Al-Dbass et al. (53) investigated the protective and therapeutic potential of N-acetyl-cysteine against PPA-induced neurotoxicity, utilizing ROC analysis to evaluate various biomarkers. This study might offer a comparative perspective on antioxidant interventions, reinforcing the relevance of luteolin in mitigating oxidative damage.

Although the current findings were based on a PPA-induced model of ASD, which primarily reflected environmental and metabolic contributions, the observed therapeutic effects of luteolin particularly its antioxidative, anti-inflammatory, and gut-protective properties suggested that it may also be beneficial in genetic models of autism. Future studies using established genetic mouse models such as *Shank3* or *Fmr1* mutants could provide further insights into the broader applicability of luteolin as a neuroprotective and disease-modifying agent in ASD.

## 5 Conclusion

This study highlighted the detrimental effects of PPA-induced oxidative stress, increased intestinal permeability, and organ dysfunction, reinforcing its role in metabolic disturbances and ASD. The findings demonstrated that luteolin effectively mitigated these effects by restoring antioxidant defenses, improving biochemical markers, and preserving gut–liver–kidney homeostasis. Its ability to modulate oxidative stress pathways and gut–organ communication underscores luteolin’s potential as both a therapeutic and protective agent. Overall, this study showed promise for the use of evidence-based dietary interventions in ASD. Future study should concentrate on luteolin’s molecular mechanisms in order to optimize its therapeutic application.

## Data Availability

The original contributions presented in the study are included in the article/supplementary material, further inquiries can be directed to the corresponding author.
